# Blood Transcriptomic Markers in Patients with Late-Onset Major Depressive Disorder

**DOI:** 10.1371/journal.pone.0150262

**Published:** 2016-02-29

**Authors:** Shigeo Miyata, Masashi Kurachi, Yoshiko Okano, Noriko Sakurai, Ayumi Kobayashi, Kenichiro Harada, Hirotaka Yamagata, Koji Matsuo, Keisuke Takahashi, Kosuke Narita, Masato Fukuda, Yasuki Ishizaki, Masahiko Mikuni

**Affiliations:** 1 Departments of Psychiatry and Neuroscience, Gunma University Graduate School of Medicine, Maebashi, Gunma, Japan; 2 Department of Molecular and Cellular Neurobiology, Gunma University Graduate School of Medicine, Maebashi, Gunma, Japan; 3 Division of Neuropsychiatry, Department of Neuroscience, Yamaguchi University Graduate School of Medicine, Ube, Yamaguchi, Japan; Kunming Institute of Zoology, Chinese Academy of Sciences, CHINA

## Abstract

We investigated transcriptomic markers of late-onset major depressive disorder (LOD; onset age of first depressive episode ≥ 50 years) from the genes expressed in blood cells and identified state-dependent transcriptomic markers in these patients. We assessed the genes expressed in blood cells by microarray and found that the expression levels of 3,066 probes were state-dependently changed in the blood cells of patients with LOD. To select potential candidates from those probes, we assessed the genes expressed in the blood of an animal model of depression, ovariectomized female mice exposed to chronic ultra-mild stress, by microarray and cross-matched the differentially expressed genes between the patients and the model mice. We identified 14 differentially expressed genes that were similarly changed in both patients and the model mice. By assessing statistical significance using real-time quantitative PCR (RT-qPCR), the following 4 genes were selected as candidates: cell death-inducing DFFA-like effector c (*CIDEC*), ribonuclease 1 (*RNASE1*), solute carrier family 36 member-1 (*SLC36A1*), and serine/threonine/tyrosine interacting-like 1 (*STYXL1*). The discriminating ability of these 4 candidate genes was evaluated in an independent cohort that was validated. Among them, *CIDEC* showed the greatest discriminant validity (sensitivity 91.3% and specificity 87.5%). Thus, these 4 biomarkers should be helpful for properly diagnosing LOD.

## Introduction

Major depressive disorder (MDD) is a highly prevalent psychiatric disorder that is associated with physical impairment, medical comorbidity, and mortality worldwide [1]. A recent study measuring the global burden of disease with disability-adjusted life-years suggested that a severe episode of MDD was a top contributor to disability among a variety of non-fatal consequences of disease and injury [2]. In addition, current treatments are only partially effective, and patients have failed to respond to trials of existing antidepressant agents [3, 4]. Heterogeneous etiology and complicated pathophysiology likely contribute to such treatment resistance. However, it is also possible that part of the ineffectiveness of antidepressant therapy for patients is a result of the clinician’s misdiagnosis and unnecessary administration of medication [5–8], as the current diagnosis of MDD is based on the evaluation of symptoms and relies on clinical interviews. Therefore, the clinician’s experience and training are essential to the appropriate treatment of MDD. Objective markers will further aid accurate diagnosis, disease classification, and outcome evaluation for MDD treatment. To date, however, there is no accepted and available objective marker, and discovery of such a marker is a top priority of research [9]. Recently, the search for biomarkers of psychiatric disorders, including MDD, has been facilitated by the examination of genes expressed in patients’ blood cells [10–14]. Blood can be obtained during a clinically relevant time window [15–18], which confers the advantage of identifying biomarkers of mood disorders [19–21].

Accumulating evidence indicates that depressive symptoms and characteristics differ by the age at onset of the first depressive episode in MDD patients, although the cut-off age is different between studies [22–29]. Patients with early-onset MDD (EOD; approximate age at onset < 25 years) demonstrate more severe depressive symptoms, higher rates of recurrence and Axis I psychiatric comorbidity, higher risk of suicide, and greater family history of mood disorders than patients with late-onset MDD (LOD; approximate age at onset > 50 years). LOD is also often associated other illnesses late in life, such as menopausal syndrome, low bone mineral density, insulin resistance, or cerebrovascular diseases rather than genetic risk [29]. The treatment response to antidepressant agents might also differ according to the age at onset in MDD patients; for instance, the antidepressant effect of escitalopram, a selective serotonin reuptake inhibitor (SSRI), is positively associated with age at onset in MDD patients with alcohol dependence [30], although conflicting results have also been reported [31]. Subtyping of elderly patients with MDD into EOD or LOD will be beneficial for psychiatric care, including adequate treatment and social support, and should alert clinicians to an increased risk of suicidality in the case of EOD. The transcriptomic marker of EOD has been reported from blood cells in young (15–19 years) patients with MDD [32]. In addition, genetic variants of AKT1 and AKT-1 binding protein have been found in LOD patients with mild cognitive impairment [33], although the transcriptomic marker of LOD has never been investigated.

The aim of the current study was to identify LOD biomarkers from the gene expression patterns of patients’ blood cells. The candidate genes were cross-matched with differentially expressed genes in the blood of an animal model of depression to select potential biomarkers because the use of transcriptomic information from animal models of psychiatric disorders has been successful in identifying potential biomarkers in human psychiatric disorders, including depression [10, 32, 34]. The discriminability of the potential candidate genes was validated in an independent cohort, and the recruitment of these subjects was conducted in an independent hospital in a different location from the first cohort.

## Material and Methods

### Participants (first cohort)

Patients with MDD who were receiving treatment at the Department of Psychiatry and Neuroscience at Gunma University Hospital were recruited. Elderly (age ≥50 years) outpatients and inpatients with MDD (n = 36) corresponding to a DSM-IV diagnosis of the melancholy type of MDD episodes were studied. Among these patients, 18 participants met the criteria for LOD (age at onset ≥50 years) with or without a remitted state and no psychiatric comorbidities, as evaluated with the Mini International Neuropsychiatric Interview (MINI) version 5, which is a structured diagnostic psychiatric interview for the DSM-IV. In addition, 4 participants (2 males and 2 females) met the criteria for EOD (age at onset < 30 years) with depressed state and no psychiatric comorbidities. All participants were recruited from March 2012 to November 2012. No participants had current endocrine disease, epilepsy, a history of head trauma with loss of consciousness, current or previous neurologic disease, a family history of hereditary neurologic disorders, or a current medical condition (e.g., active liver disease, kidney problems, or respiratory problems). Two depressed patients with LOD reached a remitted state during the study period, and they were also included as remitted patients with LOD. The depressive state was measured using the Structured Interview Guide for the Hamilton Depression (SIGH-D) rating scale. Syndromal remission was defined as a stage in which a participant did not meet the diagnosis of a MINI major depressive episode for a period of 2 consecutive months and had a SIGH-D score of less than 8.

Twelve healthy individuals were also recruited. The mean age and gender composition of the healthy individual group was matched to the groups of the LODs and EODs. Exclusion criteria for the healthy control group included a history of psychiatric illness (based on the MINI), a history of psychiatric treatment (antidepressants, anxiolytics, or antipsychotics), and a family history of psychiatric illness or treatment. The participants underwent the mini-mental state examination (MMSE) to evaluate their cognitive performance, but two patients with current LOD declined MMSE testing.

This study was performed in accordance with the Helsinki Declaration, as revised in 1989, and was approved by the Institutional Review Board of the Gunma University Hospital (Permit number: 899). All the participants received complete information on this study and signed an informed consent document.

### Participants (validation cohort)

Patients with MDD who were receiving treatment at the Department of Neuropsychiatry at Yamaguchi University Hospital were recruited. All the participants were recruited from March 2012 to August 2013. Elderly (age ≥ 50 years) outpatients and inpatients who had MDD (n = 54) with melancholic features, as defined by the DSM-IV, and no psychiatric comorbidities were studied. Among these patients, 26 participants met the criteria for LOD (age at onset ≥ 50 years) with or without a remitted state. No participants had current endocrine disease, epilepsy, a history of head trauma with loss of consciousness, current or previous neurologic disease, a family history of hereditary neurologic disorders, or a current medical condition (e.g., active liver disease, kidney problems, or respiratory problems). The depressive state was assessed using the SIGH-D. Depressed patients showed a score of greater than 18 on the SIGH-D and met the DSM-IV criteria for a major depressive episode (n = 23). The remitted patients had a score of less than 8 on the SIGH-D and met the DSM-IV criteria for full remission. Five patients who were enrolled in a depressive state reached a remitted state during the study period and were included as patients with depression in a remitted state. The participants underwent MMSE scoring, but 17 patients with current LOD declined MMSE testing.

This study was performed in accordance with the Helsinki Declaration, as revised in 1989, and was approved by the Institutional Review Board of Yamaguchi University Hospital (Permit number: H23-153). All the participants received full information on this study and signed an informed consent document before participation.

### RNA extraction from human blood

Blood samples were obtained between 9:00 and 12:00. Total RNA was extracted using a QIAmp RNA Blood Mini Kit (QIAGEN K.K., Tokyo, Japan) according to the manufacturer’s instructions. The eluted samples were processed with the standard method of ethanol precipitation, and the RNA pellet was dissolved in RNase-free water. The RNA quantity and quality were assessed using a NanoDrop ND-1000 spectrophotometer (Thermo Fisher Scientific Inc., Waltham, MA, USA) and an Agilent Bioanalyzer (Agilent Technologies, Palo Alto, CA, USA) as recommended.

### Animals

Female C57BL/6J mice (8 weeks of age) were purchased from Charles River Laboratories Japan, Inc. (Kanagawa, Japan). The animals were housed with 4–6 mice per cage (16.5 × 27 × 12.5 (H) cm) and had free access to food and water. The animal room was maintained at 22 ± 3°C with a 12-h light-dark cycle (lights on at 6:00, lights off at 18:00). The mice were acclimatized to the laboratory environment for 1 week and then were bilaterally ovariectomized (OVX) or underwent a sham operation under sodium pentobarbital (50 mg/kg, i.p.) anesthesia. All behavioral observations and blood samplings were performed between 10:00 and 16:00 each day. This study was performed in accordance with the Guidelines for Animal Experimentation at Gunma University Graduate School of Medicine and were approved by the Gunma University Ethics Committee (Permit number: 12–006). Every effort was made to minimize the number of animals used, and their suffering.

### Chronic ultra-mild stress (CUMS) procedure

Two weeks after the OVX surgery, the CUMS procedure was initiated. The mice were exposed to CUMS for 6 weeks in accordance with our previous report [35]. Three stressors were used in this study. For the first stressor, two of five diurnal stressors were delivered over a 1-h period in the morning and over a 2-h period in the evening, with a 2-h stress-free period between the two stressors. The five diurnal stressors included cage tilt (45°), small cage restriction (9.5 cm × 17 cm × 10.5 (H) cm), switching to the home-cage of another group, a soiled cage (50 ml of water in sawdust bedding), and odor (50% acetic acid). The second stressor consisted of four nocturnal stressors applied between 16:00 h and 10:00 h, including one overnight period with difficult access to food, one overnight period with the lights on, one overnight period with a 45° cage tilt, and one overnight period in a soiled cage. For the third stressor, a reversed light/dark cycle was used from Friday evening to Monday morning. This procedure was scheduled over a 1-week period and was repeated six times. The non-stressed mice were handled weekly to clean the sawdust bedding. The health of animals were monitored weekly in non-stressed mice and every weekday in stressed mice. There was no unexpected death in this study.

### Behavioral tests of mice

One day after CUMS cessation, the following behavioral tests were conducted. A forced swimming test was administered in which each mouse was placed in an acrylic cylinder (22 cm in height, 11.5 cm in diameter) containing 15 cm of water at room temperature (22 ± 3°C). The cylinder was placed in an isolation box. The behavior of each mouse was recorded for 5 min using a CCD camera connected to a personal computer and analyzed using ImageJ PS1 (O’Hara & Co., Ltd., Tokyo, Japan), which is a modified software package that is based on the public domain ImageJ program (developed at the U.S. National Institutes of Health and available at http://rsb.info.nih.gov/ij).

An open-field test was administered in which each mouse was placed in the center of an open-field apparatus (50 cm × 50 cm × 40 (H) cm) that was illuminated by light-emitting diodes (30 lux at the center of the field) and allowed to move freely for 5 min. The time spent in the central area of the field (36% of the field) was recorded as the index of interest. The data were collected and analyzed using ImageJ OF4 (O’Hara & Co., Ltd.), which is modified software that is also based on the public domain ImageJ program.

The mice were also administered an elevated plus-maze test in which each mouse was placed onto the central platform so that it faced an enclosed arm. The observation started immediately thereafter and lasted for 5 min. The apparatus consisted of a plus-shaped maze that was elevated 40 cm from the floor and that was composed of two open arms (25 cm × 5 cm) and two enclosed arms (25 cm × 5 cm) that were arranged so that the arms of the same type were opposite each other. The behavioral testing was conducted in a darkened, sound-proof room, and the maze was lit obliquely from above to provide 15 lux to every part of the maze. The number of whole body entries (all four paws) made into each arm and the total path length were recorded automatically and analyzed using ImageJ EP (O’Hara & Co., Ltd.). The total path length and the percentage of time spent in the open arms were calculated.

### RNA extraction from mouse blood

Mouse blood (300 μl) was collected under pentobarbital anesthesia (50 mg/kg, i.p.) via the vena cava between 9:00 and 12:00. Then, the brain was also obtained, but it was used for subsequent study. The blood was immediately heparinized and centrifuged (1,000 ×*g*, 2 min). The total RNA in the pellet was extracted using the GeneJet Whole Blood RNA Purification Mini Kit (Thermo Fisher Scientific Inc.) according to the manufacturer’s instructions. The RNA quantity and quality were determined using a NanoDrop ND-1000 spectrophotometer (Thermo Fisher Scientific Inc.) and an Agilent Bioanalyzer (Agilent Technologies) as recommended.

### Microarray

The total RNA was amplified and labeled with Cyanine 3 (Cy3) using an Agilent Low Input Quick Amp Labeling Kit, one-color (Agilent Technologies), according to the manufacturer’s instructions. Briefly, 100 ng of total RNA was reverse-transcribed to double-stranded cDNA using a poly dT-T7 promoter primer. The primer, template RNA, and quality-control transcripts of known concentration and quality were first denatured at 65°C for 10 min and then incubated for 2 h at 40°C with 5× first-strand buffer, 0.1 M dithiotreitol, 10 mM dNTP mix, and AffinityScript RNase Block Mix. The AffinityScript enzyme was then inactivated at 70°C for 15 min. The cDNA products were used as templates for the in vitro transcription to generate fluorescent cRNA. The cDNA products were mixed with a transcription master mix in the presence of T7 RNA polymerase and Cy3-labeled CTP and incubated at 40°C for 2 h. The labeled cRNAs were purified using QIAGEN’s RNeasy mini spin columns and eluted in 30 μl of nuclease-free water. After amplification and labeling, the cRNA quantity and cyanine incorporation were determined using a NanoDrop ND-1000 spectrophotometer and an Agilent Bioanalyzer.

For each hybridization, 600 ng of Cy3-labeled cRNA was fragmented and hybridized at 65°C for 17 h to the Agilent SurePrint G3 Human GE 8×60K v2 Microarray (Design ID: 039494) and the Agilent SurePrint G3 Mouse GE 8×60K Microarray (Design ID: 028005). After washing, the microarrays were scanned using an Agilent DNA microarray scanner.

The intensity values of each scanned feature were quantified using Agilent feature extraction software version 10.7.3.1, which performs background subtractions. Normalization was performed using Agilent GeneSpring GX version 11.0.2 (per chip: normalization to the 75^th^ percentile shift; per gene: none). The probes that were declared as “present” in all the assayed samples and that displayed a raw intensity value above 50 in at least 2 samples were used for the following statistical analyses.

Information concerning our data was submitted to the Gene Expression Omnibus, with accession numbers GSE76826 (human) and GSE72262 (mouse).

### Real-time quantitative PCR (RT-qPCR)

The expression level of each candidate gene in the microarray was confirmed using RT-qPCR. The cDNA was synthesized using a PrimeScript RT reagent kit (Takara Bio Inc., Shiga, Japan) with oligo dT primers and 500 ng of total RNA. The synthesized cDNA was mixed with SYBR Premix Ex Taq II (Takara Bio Inc.) and specific primers. Amplification was performed with 50 cycles, each at 95°C for 3 s and at 60°C for 30 s, in an RT-qPCR system (StepOnePlus Real-Time PCR System, Applied Biosystems Japan Ltd., Tokyo, Japan). We chose ribosomal protein S29 (*RPS29*) as the reference gene [36]. Sequences of the primers used for PCR are listed in S1 Table.

### Statistics

Significant differences in the demographic data from the first cohort were analyzed by one-way analysis of variance (ANOVA) with a Bonferroni multiple comparison test. Significant differences between two groups in the validation cohort were determined with an unpaired *t*-test (Student’s *t*-test or Welch’s *t*-test). Differences in sex composition were analyzed using Fisher’s exact test. Statistical significance was defined as a *p*-value less than 0.05.

For the microarray data in humans, the differences in gene expression between the groups were determined by principal component analysis (PCA) and one-way ANOVA. In the one-way ANOVA, a Benjamini-Hockberg false discovery rate (FDR) less than 0.05 was considered significant. Differentially expressed genes between groups were determined by a post-hoc Tukey’s honestly significant difference (HSD) test (*p* < 0.05).

In the RT-qPCR, the amounts of the target genes were calculated with ΔΔcycle threshold (Ct) analysis. Significant differences were determined by one-way ANOVA followed by a post-hoc Tukey’s HSD test in the first cohort and by an unpaired *t*-test (Student’s *t*-test or Welch’s *t*-test) in the validation cohort. The difference between the Ct value of the target gene and the control gene in RT-qPCR, i.e., the ΔCt value, was used as an individual parameter to determine the receiver-operating characteristic (ROC) curves. The highest Youden index and the area under the curve were calculated from the ROC curves for each gene, and the discriminatory cut-off points were determined. Sensitivity represented the true positive rate in the group of depressed patients with LOD in the data set. Specificity represented the true negative rate in the non-depressed group.

Significant differences in animal behaviors were determined by two-way ANOVA followed by a Bonferroni test. For the microarray data in animals, the factorial effects on gene expression levels were determined by two-way ANOVA (Benjamini-Hockberg FDR < 0.05). One-way ANOVA (Benjamini-Hockberg FDR < 0.05) followed by Tukey’s post-hoc HSD test (*p* < 0.05) was conducted to determine the differentially expressed genes specific to OVX+CUMS mice.

## Results

The demographic data of the first cohort study are shown in Table 1. No significant difference was found in the mean age, sex composition, or MMSE scores between the depressed patients with LOD (DPs; n = 10), the remitted patients with LOD (RMs; n = 10), and the healthy controls (HCs; n = 12). The SIGH-D scores were significantly higher in the DPs than in the HCs and RMs. The age at onset did not differ between the DPs and RMs.

**Table 1:** Demographic data of the participants in the first cohort.

	Age (y)	Age at onset (y)	Sex (M/F)	SIGH-D	MMSE
HC	62.5 ± 2.7	-	5/7	3.3 ± 0.6	27.8 ± 0.9
DP	69.3 ± 4.0	61.0 ± 2.9	4/6	23.4 ± 3.3^a^	26.5 ± 0.9
RM	73.6 ± 3.4	64.1 ± 2.9	5/5	3.3 ± 0.5^b^	27.8 ± 0.6

The data represent the means ± SE.

^a^*p* < 0.01 vs. HC group.

^b^*p* < 0.01 vs. DP group (Bonferroni test).

**Fig 1:**
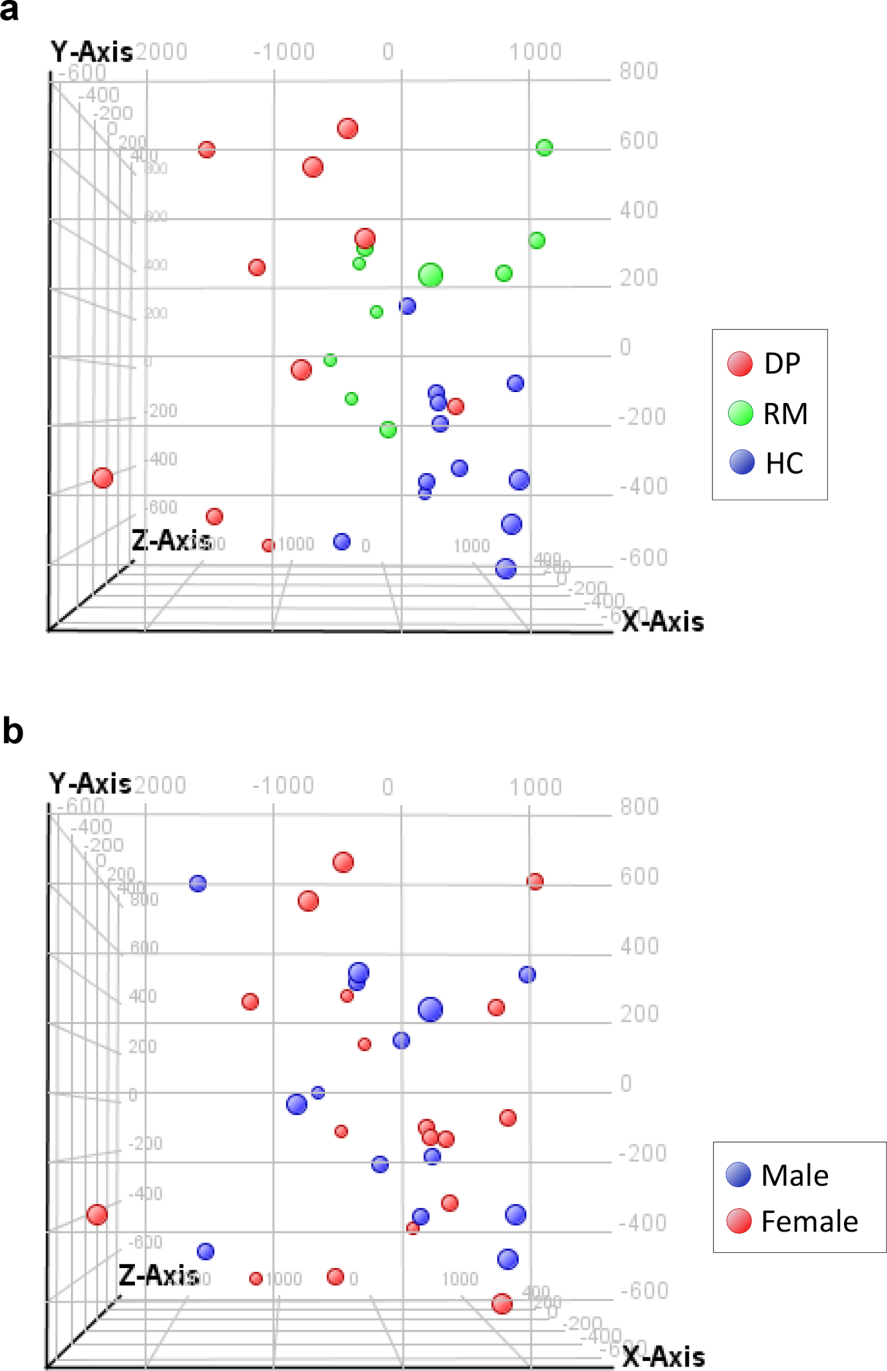
Gene expression profiles in humans in the first cohort. A total of 23,671 probes were examined in human blood cells. The PCA of the data set revealed that the first principal component (x-axis) explained 57.71% of the differentially expressed genes, the second principal component (y-axis) explained 26.15%, and the third principal component (z-axis) explained 16.14%. Each dot corresponds to an individual PCA score and is colored according to the state (a) and sex (b).

The genome-wide expression of mRNA in blood cells was studied with microarray analysis, and 23,671 detected probes were scored with PCA. The distributions of the PCA scores in the DPs, RMs, and HCs were fairly well separated (Fig 1A). The distributions of the PCA scores in the two sexes were largely similar (Fig 1B). One-way ANOVA with multiple comparison analysis revealed that the expression of 3,066 probes was significantly different in the DPs compared with the HCs and in the RMs compared with the DPs, but not in the RMs compared with the HCs, indicating that the expression of these genes was state-dependently changed (S2 Table).

To select the potential candidate biomarkers from the 3,066 probes, we next cross-matched the differentially expressed genes in the blood samples from the animal model of depression because use of transcriptomic data in animal models of depression have successfully identified potential biomarkers in human depression [10, 32]. To our knowledge, however, there is no accepted animal model of LOD or animal model of depression using elder rodents. Instead of an LOD model, OVX female rodents are widely used as a model of menopause [37, 38], and OVX mice exposed to sub-chronic stress demonstrate abnormalities in several behavioral tasks similar to animal models of depression [39, 40], which may reflect the fact that MDD is approximately twice as prevalent among women compared with men [41]. Indeed, female patients with MDD in menopause would be included in the current LOD category. Therefore, in this study, OVX mice exposed to the CUMS (OVX+CUMS mice) were used as a tool to select potential candidate biomarkers.

Before the examination of gene expression in mice, we used several behavioral tests to validate the induction of emotional changes caused by OVX and CUMS. The schedule of CUMS is shown in Table 2.

**Table 2:** Weekly schedule of the CUMS protocol.

	Mon.	Tue.	Wed.	Thu.	Fri.	Sat.	Sun.
**10:00–11:00 (1h)**	small cage	cage switch	cage tilt	order	small cage	reversed light/dark cycle	reversed light/dark cycle
**13:00–15:00 (2h)**	order	soiled cage	small cage	cage switch	cage tilt	reversed light/dark cycle	reversed light/dark cycle
**16:00–9:00 (overnight)**	difficult access to food	light on	cage tilt	soiled cage	reversed light/dark cycle	reversed light/dark cycle	reversed light/dark cycle

The CUMS exposure was started 2 weeks after the OVX surgery. The weekly schedule of CUMS was repeated six times. One day after CUMS cessation, the behavioral tests and the blood sampling were conducted.

**Fig 2:**
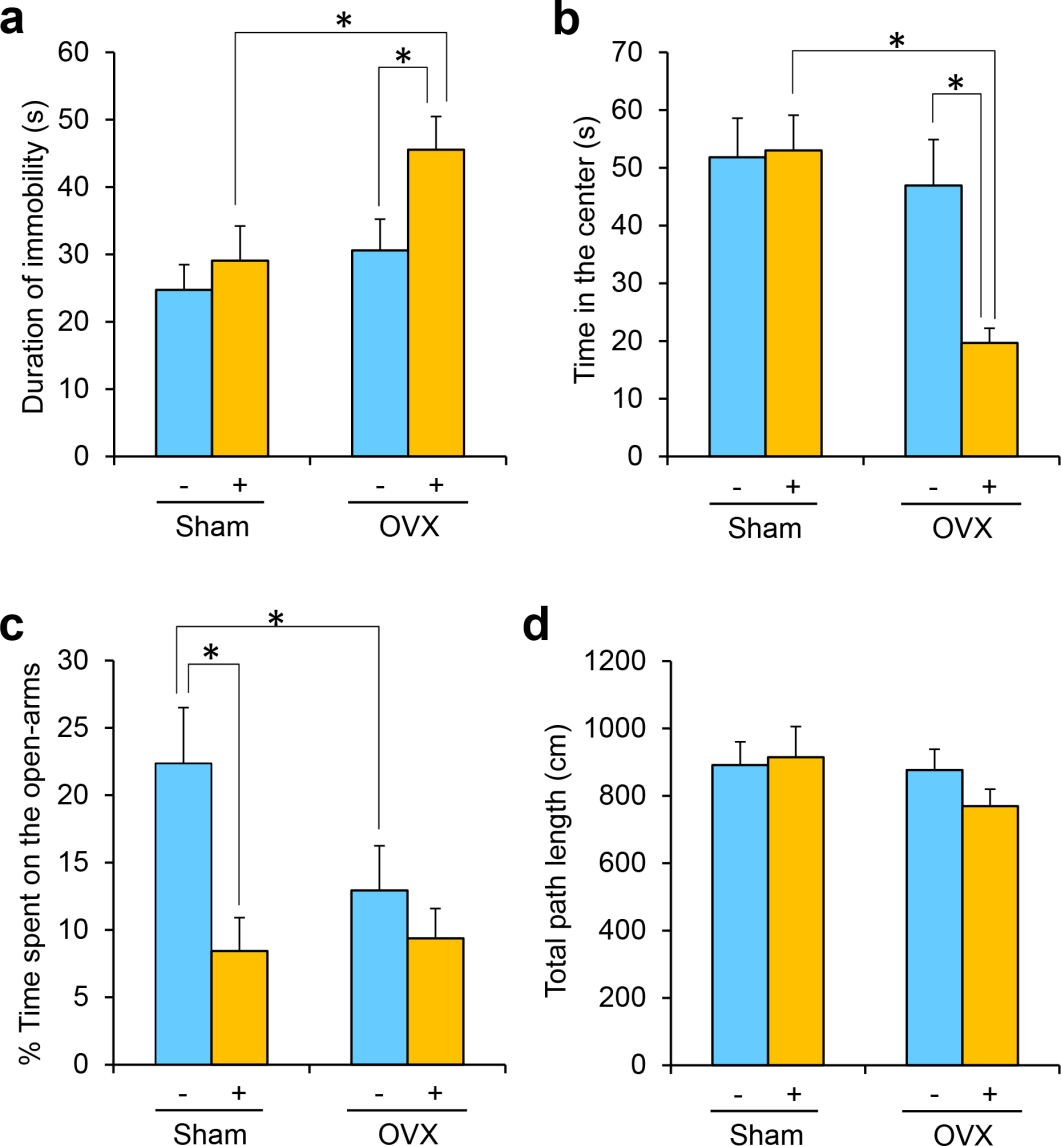
Emotional behaviors in the model mice. (a) The duration of immobility in the forced swimming test (n = 24 per group). (b) The time spent in the center field in the open-field test (n = 9 non-stressed sham mice, n = 8 non-stressed OVX mice, n = 8 sham+CUMS mice, and n = 9 OVX+CUMS mice). (c) The percentage of time spent on the open-arms, and (d) the total distance traveled in the elevated plus-maze test (n = 10 non-stressed sham mice, n = 10 non-stressed OVX mice, n = 9 sham+CUMS mice, and n = 10 OVX+CUMS mice). Non-stressed groups are represented by “—”, and CUMS-treated groups are represented by “+”. The data are displayed as the means + SEs. Significant differences between the groups are represented by “*” (*p* < 0.05, post-hoc Bonferroni test).

In the forced swimming test, a two-way ANOVA showed significant effects of CUMS [F(1, 92) = 4.314; *p* < 0.05] and OVX [F(1, 92) = 5.779; *p* < 0.05], but not a CUMS×OVX interaction [F(1, 92) = 1.304; *p* = 0.26]. Post-hoc comparisons revealed that CUMS significantly increased the duration of immobility in mice when the mice were subjected to OVX (Fig 2A). In the open-field test, a two-way ANOVA revealed that the time spent in the center was significantly affected by CUMS [F(1, 30) = 4.614; *p* < 0.05], OVX [F(1, 30) = 9.896; *p* < 0.01] and the CUMS×OVX interaction [F(1, 30) = 5.476; *p* < 0.05]. Post-hoc comparisons revealed that CUMS significantly decreased the time spent in the center when the mice were subjected to OVX (Fig 2B). In the elevated plus-maze test, a two-way ANOVA revealed that the time spent in the open-arms was significantly affected by CUMS [F(1, 35) = 7.601; *p* < 0.01], but not by OVX [F(1, 35) = 1.791; *p* = 0.19] or the CUMS×OVX interaction [F(1, 35) = 2.680; *p* = 0.11]. Post-hoc comparisons revealed that OVX significantly decreased the time spent in the open-arms in non-stressed mice (Fig 2C). In addition, CUMS significantly decreased the time spent in the open-arms in sham-operated mice (Fig 2C). The total distance traveled by mice in the elevated plus-maze was neither affected by CUMS [F(1, 35) = 0.376; *p* = 0.54], OVX [F(1, 35) = 1.369; *p* = 0.25] nor the CUMS×OVX interaction [F(1, 35) = 0.901; *p* = 0.35] (Fig 2D). These results indicate that OVX mice exhibited marked emotional changes when they were subjected to the CUMS.

The expression of mRNA in the blood samples of these four groups of mice was studied using microarray analysis (n = 6 in each group). In total, 23,561 probes were detected in the blood of mice. Among them, two-way ANOVA revealed that the expression levels of 12,536 probes and 10,917 probes were significantly altered by OVX and CUMS, respectively. Only 10 probes were significantly altered in their expression levels by the CUMS×OVX interaction. Because the effect of the CUMS×OVX interaction on the expressed gene levels in the blood of mice was small, and we did not find the candidate gene from the state-dependent 3,066 probes, we next determined the differentially expressed genes in the OVX+CUMS mice (but not in the non-stressed OVX mice or the sham-operated CUMS-treated mice). As a result, the expression levels of 637 probes were significantly changed in the OVX+CUMS mice, but not in the non-stressed OVX mice or the sham-operated CUMS-treated mice, compared with the control (sham-operated non-stressed) mice. The differentially expressed probes are listed in S3 Table.

**Fig 3:**
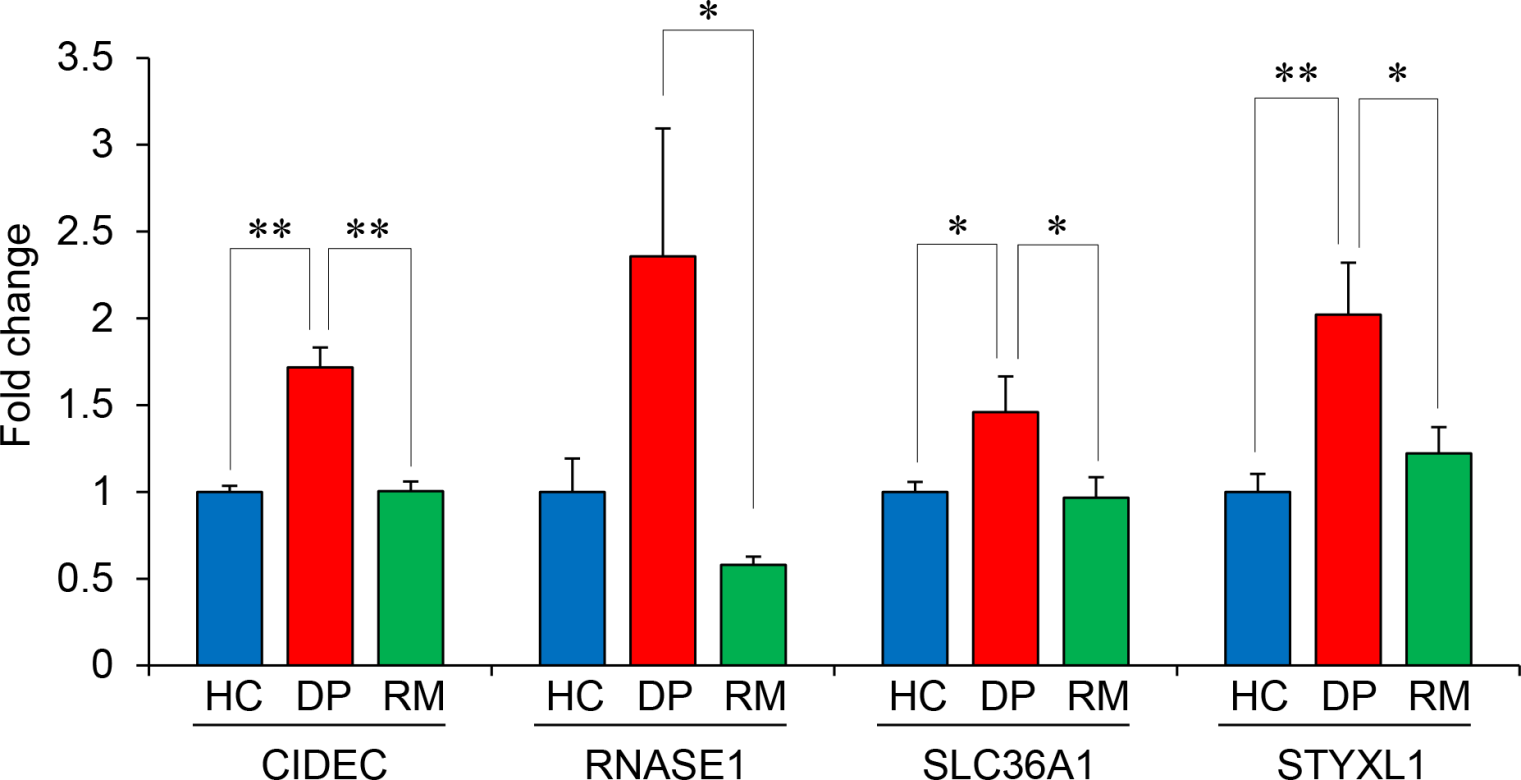
Quantitative analysis of *CIDEC*, *RNASE1*, *SLC36A1*, and *STYXL1* mRNA expression in the first cohort. The data represent the means + SEs. *p < 0.05, **p < 0.01 between the indicated groups (Tukey’s HSD test).

The differentially expressed genes in the patients (3,066 state-dependent probes) were cross-matched with those identified in the animal model (637 OVX+CUMS-specific probes) by reference to the gene symbols, and 14 genes were identified. We quantitatively measured the expression levels of these 14 genes with RT-qPCR in the human samples. The mRNA levels of cell death-inducing DFFA-like effector c (*CIDEC*), solute carrier family 36 member-1 (*SLC36A1*), and serine/threonine/tyrosine interacting-like 1 (*STYXL1*) were significantly increased in the DPs compared with the HCs, and these effects were completely reversed in the RMs (Fig 3). The mRNA levels of ribonuclease 1 (*RNASE1*) were increased, although not significantly, in the DPs compared with the HCs. However, the *RNASE1* levels were significantly decreased in the RMs compared with the DPs (Fig 3). The other genes did not show state-dependent changes (S1 Fig). Thus, we selected *CIDEC*, *RNASE1*, *SLC36A1*, and *STYXL1* as candidate state-dependent biomarkers of LOD.

To evaluate the discriminability of these candidates, a validation cohort study was conducted in the Department of Neuropsychiatry at Yamaguchi University Hospital, which was a different location from that of the first cohort study. The mean age, age at onset, sex composition, and MMSE score did not differ between the DPs and the RMs (Table 3). The SIGH-D scores were significantly lower in the RMs than in the DPs (Table 3).

**Table 3:** Demographic data of the participants in the validation cohort.

	Age (y)	Age at onset (y)	Sex (M/F)	SIGH-D	MMSE
**DP**	60.9 ± 1.4	56.8 ± 1.2	9/14	24.2 ± 1.0	27.3 ± 1.1
**RM**	59.6 ± 2.7	56.3 ± 2.8	2/6	1.5 ± 0.9^a^	28.3 ± 0.8

The data represent the means ± SE.

^a^*p* < 0.01 vs. DP group (unpaired *t*-test).

**Fig 4:**
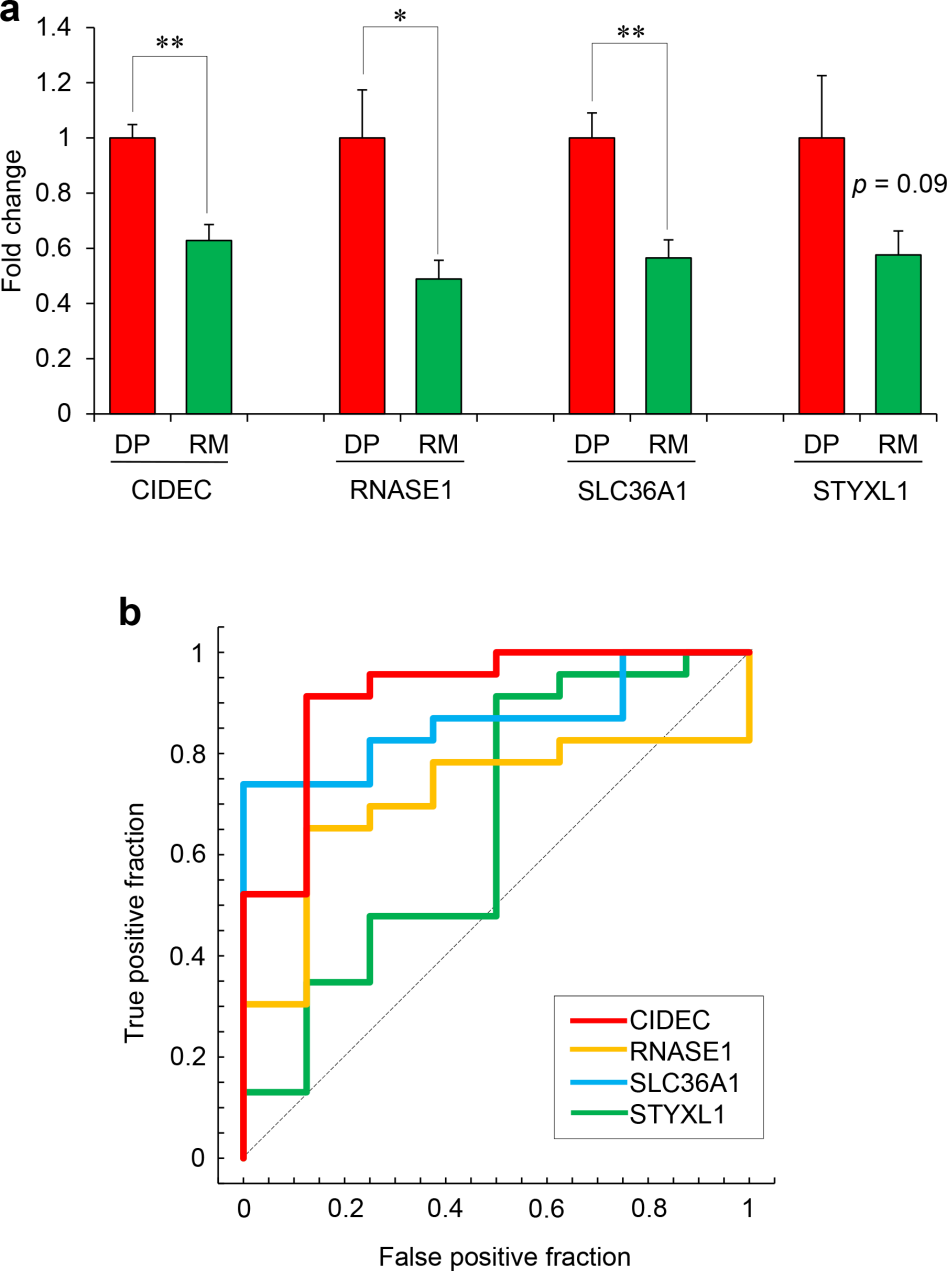
Quantitative analysis of 4 biomarker genes in the validation cohort. (a) The expression levels of the four putative biomarkers in the validation cohort. The data represent the means + SE. *p < 0.05, **p < 0.01 between the indicated groups (Tukey-HSD test). (b) The ROC curves for the ΔCt values of *CIDEC*, *RNASE1*, *SLC36A1*, and *STYXL1* in the validation cohort.

RT-qPCR showed that the expression levels of the 4 genes were higher in the DPs than in the RMs, with statistically significant differences for *CIDEC*, *RNASE1*, and *SLC36A1*, but not for *STYXL1* (*p* = 0.09) (Fig 4A). These results were largely consistent with the results from the first cohort study. ROC curves were created using the data set from the validation cohort. The greatest area under the ROC curve (AUC) was obtained for the expression levels of *CIDEC*, followed by *SLC36A1*, *RNASE1*, and *STYXL1* (Fig 4B).

Furthermore, the expression levels of *CIDEC* showed the highest discriminability for depressed vs. non-depressed states (sensitivity 91.3%; specificity 87.5%). The values of each ROC curve in the validation cohort are summarized in Table 4.

**Table 4:** The values of each ROC curve in the validation cohort study.

	AUC	Cut-off points	Sensitivity (%)	Specificity (%)
***CIDEC***	0.9185	10.1031	91.3	87.5
***RNASE1***	0.7120	13.7514	65.2	87.5
***SLC36A1***	0.8641	9.8859	73.9	100.0
***STYXL1***	0.6576	13.8266	91.3	50.0

ΔCt values were used as an individual parameter to determine the ROC curves. The AUC and the highest Youden index (cut-off point) are calculated from the ROC curves in each gene. Sensitivity and specificity represent the true positive rate and the true negative rate, respectively.

We assessed the expression levels of the four genes in the blood cells of elder patients with EOD in the first cohort. The mean age (57.0 + 2.35 y) and MMSE scores (28.8 + 1.25) of the EODs were not different from the HCs (see Table 1). The SIGH-D scores of EODs (27.3 + 4.8) was significantly higher than the HCs (*p* < 0.05, unpaired *t*-test). RT-qPCR showed that the expression levels of the 4 genes were not different between the EODs and the HCs (S2 Fig).

## Discussion

In the current PCA, we found that the gene expression patterns in the blood cells of DPs were largely different from those in the blood cells of HCs. In addition, the gene expression patterns in the RMs was largely different from those in the DPs, but resembled that in the HCs. These results indicate that the gene expression patterns in blood cells reflect the depressive state in LOD. Depression in elder patients is a risk factor for developing dementia [29, 42]. However, the MMSE scores in the current DPs were not different from those of the HCs and RMs. Therefore, the current transcriptomic data represent the depressive state and unlikely show the influence of cognitive impairment.

In this study, we used the transcriptomic data in the blood of OVX+CUMS mice as a tool to select potential candidates from the 3,066 state-dependent probes in the patients. Before the microarray examination, we evaluated the behavioral abnormalities in the current OVX+CUMS mice and found that the OVX+CUMS mice demonstrated depression-like behavior in the forced swimming test and anxiety-like behaviors in the open-field test and the elevated plus-maze test, which are observations consistent with previous reports [39, 40]. By the microarray analysis, the expression of 637 probes was specifically altered in the blood of OVX+CUMS mice. By cross-matching between the state-dependent 3,066 genes in the human data set and the differentially expressed 637 genes in the model mice, we found 14 genes that were similarly changed between the patients and the model mice. Among them, the expression levels of *CIDEC*, *RNASE1*, *SLC36A1*, and *STYXL1* mRNA were significantly changed depending on the state of depression in the RT-qPCR analysis, which was consistent with the results of the microarray analysis. It is important to note that the high discriminability of these 4 genes into depressed or non-depressed states in patients with LOD was validated in the independent cohort. Based on these results, we propose that *CIDEC*, *RNASE1*, *SLC36A1*, and *STYXL1* mRNA expression levels in blood cells constitute state-dependent biomarkers of LOD. In particular, *CIDEC* may be the best biomarker because it showed the highest ability to determine an individual’s depression state. We compared the current 4 genes with the top-ranked differentially expressed genes in the published studies. The current 4 genes are different from the previous ones. However, *SLC36A1* mRNA was involved in the list of differentially expressed genes in comparison between MDD patients and healthy controls [43] and STYXL1 mRNA was involved in the list of differentially expressed genes in MDD patients in comparison between responders and non-responders to the antidepressant therapy [44]. In addition, the current 4 genes did not discriminate the elder patients with EODs from the HCs in this study and are different from the EOD markers previously reported [32]. Therefore, the gene expression patterns in blood cells in MDD may differ according to the age at onset of MDD, and such cases may be separated in EOD or LOD by measuring transcriptomic markers.

The pathophysiological roles of these biomarker proteins have not been determined in MDD and have only been investigated in other diseases. CIDEC is a member of the cell death-inducing DNA fragmentation factor-α-like effector family, which was initially identified as a group of factors that induce apoptosis [45, 46]. Recent studies suggest that this class of protein is closely related to energy balance and obesity. CIDEC is highly expressed in adipose tissues in humans and mice [47]. In addition, the CIDEC protein is localized around lipid droplets in adipocytes and plays an important role in lipid droplet formation [47]. In human adipocytes, insulin up-regulates *CIDEC* mRNA expression mediated by the phosphatidylinositol 3-kinase—c-Jun N-terminal kinase 2 pathway [48]. CIDEC-deficient mice appear to be largely protected from the obesity, hyperleptinemia, glucose intolerance and insulin resistance induced by a high-fat diet [49]. However, the mechanism that regulates *CIDEC* transcriptional activity in blood mononuclear cells remains unknown.

RNASE1 is the secretory/pancreatic type of ribonuclease. Although RNASE1 produced in the pancreas and excreted into the gut seems to degrade the large amounts of RNA in food [50], the enzyme is known to be present in nondigestive tissues other than the pancreas, such as the brain [51], kidney [52], and venous endothelial cells [53], suggesting other in vivo functions. RNASE1 in vascular endothelial cells is released by prothrombotic or pro-inflammatory stimuli and degrades extracellular RNAs, which stimulate pro-inflammatory activities [54, 55]. Furthermore, the *RNASE1* mRNA level in peripheral mononuclear cells is 5-fold higher in patients with heart failure than in controls [56].

SLC36A1, which is also known by the names proton-coupled amino acid transporter 1 and lysosomal amino acid transporter 1, functions as a H+-coupled amino acid symporter [57]. SLC36A1 is expressed at the luminal surface of the small intestine but is also commonly found in lysosomes in many cell types, including neurons, suggesting that it is a multipurpose carrier with distinct roles in different cells. A broad range of amino acids and many orally active amino acid-based drugs and derivatives, such as a large number of γ-aminobutyric acid-related and proline-related compounds, are transported by SLC36A1 as its substrates. By contrast, tryptophan derivatives such as serotonin and 5-hydroxy-L-tryptohpan are non-transported competitive inhibitors of SLC36A1 [57]. Furthermore, a selective serotonin reuptake inhibitor, sertraline, inhibits SLC36A1 activity [58]. In addition, the *SLC36A1* mRNA level is increased in the jejunum of high fat diet-fed mice [59], and the transcriptional activity of *SLC36A1* is up-regulated by insulin in skeletal muscle cells [60].

STYXL1 is a pseudo-phosphatase member of the dual-specificity phosphatase subfamily of the protein tyrosine phosphatases. STYXL1 is catalytically inactive due to the absence of two amino acids from the signature motif that are essential for phosphatase activity. STYXL1 is required for intrinsic apoptosis. Knockdown of STYXL1 provides a specific block during mitochondrion-dependent apoptosis by blocking cytochrome c release, allowing cells to survive high doses of chemotherapeutics [61]. Recently, a homozygous missense mutation in *STYXL1* was identified in patients with moderate intellectual disability accompanied with seizures and behavioral complexities, and the expression level of *STYXL1* mRNA in the EBV-transformed lymphoblastoid cells in those patients was decreased [62].

We acknowledge several general limitations to our experimental design. First, our results are limited by the small size of the cohort and require validation in a larger cohort of controls and patients with LOD, including those in remission. However, although the sample size of this study was small, the number of differentially expressed genes (over 3,000 probes) is larger than that in previous blood-based gene expression studies in patients with MDD [10, 16, 19]. Because MDD is thought to be clinically and genetically heterogeneous, the current classification (LOD without cognitive impairment) may mask the individual variability in expressed genes in blood cells and may lead to the detection of a large number of differentially expressed genes in the microarray. Follow-up studies are also required to demonstrate reproducible results and to evaluate the compatibility of the microarray with other types of depression. In this study, we used OVX+CUMS mice as the animal model of depression because, to our knowledge, an animal model of LOD has never been developed. However, it should be noted that the current study *does not* suggest that OVX+CUMS mice represent an animal model of LOD.

## Conclusions

We identified putative state-dependent biomarkers of LOD, *CIDEC*, *RNASE1*, *SLC36A1*, and *STYXL1* mRNA, in blood cells. These 4 biomarkers should be helpful for properly diagnosing LOD.

## Supporting Information

S1 Fig The expression levels of 10 biomarker candidates in human samples assessed with RT-qPCR. The RNA samples extracted from the white blood cells of the HCs (n = 12), DPs (n = 10), and RMs (n = 10) were used. The data are the means + SE. *p < 0.05 and **p < 0.01 between the indicated groups (Tukey-HSD test). BANP; BTG3 associated nuclear protein, RAB11FIP4; RAB11 family interacting protein 4 (class II), ARFRP1; ADP-ribosylation factor related protein 1, BCL11B; B-cell CLL/lymphoma 11B (zinc finger protein), FYCO1; FYVE and coiled-coil domain containing 1, NIPAL3; NIPA-like domain containing 3, RPL23A; ribosomal protein L23a, RPS2; ribosomal protein S2, SIGIRR; single immunoglobulin and toll-interleukin 1 receptor (TIR) domain, SLC35F2; solute carrier family 35, member F2.
(TIF)

S2 Fig Quantitative analysis of CIDEC, RNASE1, SLC36A1, and STYXL1 mRNA expression in depressed patients with EOD. The data are the means of ΔCt values + SE. No significant difference between groups (Tukey-HSD test).
(TIF)

S1 Table Primer pairs are listed alphabetically. (PDF)

S2 Table List of differentially expressed 3,066 probes. (XLSX)

S3 Table List of differentially expressed 637 probes. We detected significant differences in expression in the WB of OVX+CUMS mice, but not in the non-stressed OVX mice or the sham-operated CUMS mice, compared with the control (sham-operated non-stressed) mice (one-way ANOVA followed by Tukey’s HSD test).
(XLSX)
