# Supplementary figures and images for: Blood Transcriptomic Markers in Patients with Late-Onset Major Depressive Disorder
Source: PLoS One. 2016 Feb 29;11(2):e0150262. doi: 10.1371/journal.pone.0150262 (PMC4771207; doi:10.1371/journal.pone.0150262)

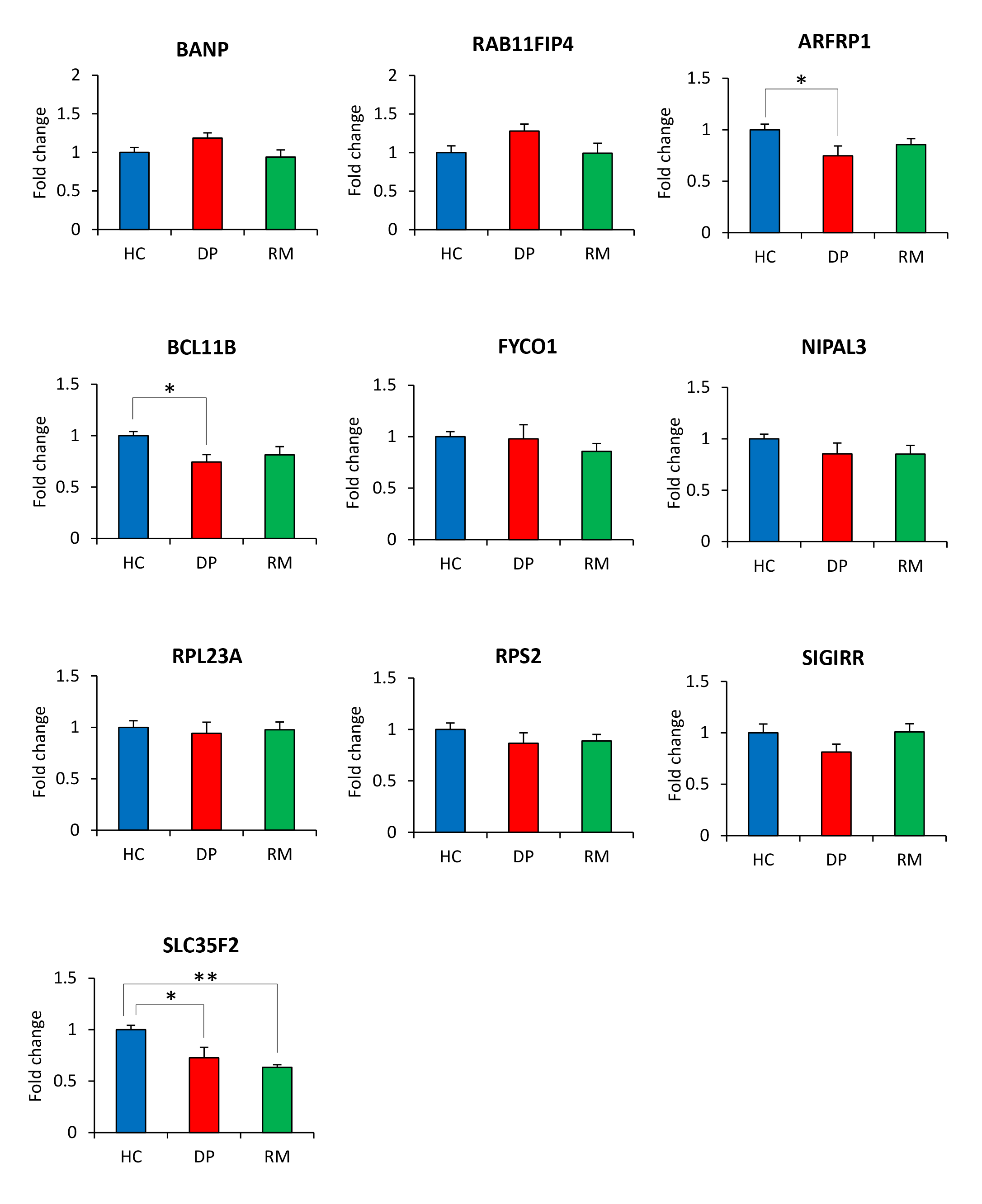

Supplement: S1 Fig — The RNA samples extracted from the white blood cells of the HCs (n = 12), DPs (n = 10), and RMs (n = 10) were used. The data are the means + SE. *p < 0.05 and **p < 0.01 between the indicated groups (Tukey-HSD test). BANP; BTG3 associated nuclear protein, RAB11FIP4; RAB11 family interacting protein 4 (class II), ARFRP1; ADP-ribosylation factor related protein 1, BCL11B; B-cell CLL/lymphoma 11B (zinc finger protein), FYCO1; FYVE and coiled-coil domain containing 1, NIPAL3; NIPA-like domain containing 3, RPL23A; ribosomal protein L23a, RPS2; ribosomal protein S2, SIGIRR; single immunoglobulin and toll-interleukin 1 receptor (TIR) domain, SLC35F2; solute carrier family 35, member F2. (TIF) [file pone.0150262.s001.tif]

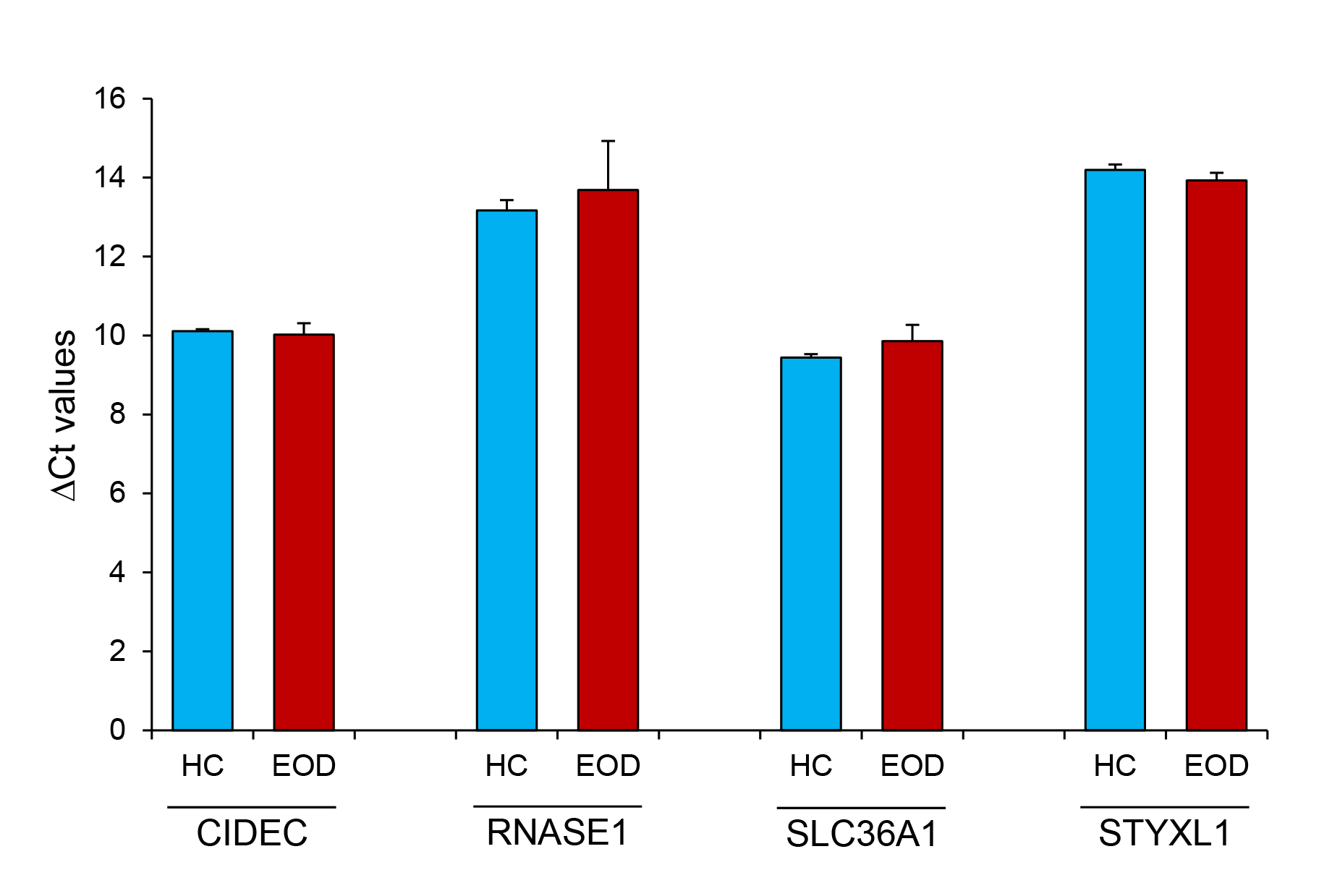

Supplement: S2 Fig — The data are the means of ΔCt values + SE. No significant difference between groups (Tukey-HSD test). (TIF) [file pone.0150262.s002.tif]
