# Supplementary material for: Blood Transcriptomic Markers in Patients with Late-Onset Major Depressive Disorder
Source: PLoS One. 2016 Feb 29;11(2):e0150262. doi: 10.1371/journal.pone.0150262 (PMC4771207; doi:10.1371/journal.pone.0150262)
Supplement: S1 Table — (PDF) [file pone.0150262.s003.pdf]

Gene symbol	Primer sequence		Amplicon size (bp)	Accession number
ARFRP1	Forward	TGCATCGGGAGGCTTCAGAG	125	NM_001134758
	Reverse	CCACTCCCAACTCACAGGGG		
BANP	Forward	CGGGGCCATCCAGATTCAGT	147	NM_017869
	Reverse	GCCTTCAGACGCACTTCAGC		
BCL11B	Forward	TGCACTCTTTCCTCCTTGCCA	141	NM_138576
	Reverse	GGGGCATCCAAAGGCAACAG		
CIDEC	Forward	CTGGAAACCTGGGGTGGGTT	91	NM_022094
	Reverse	CTGGATGGGCTAAGCTGCCT		
FYCO1	Forward	CGGGGCCATCCAGATTCAGT	124	NM_024513
	Reverse	GCCTTCAGACGCACTTCAGC		
NIPAL3	Forward	CGGTCTCCTTTTGAGCAGCC	123	NM_020448
	Reverse	CCCAGCGTTCAACTCCACAC		
RAB11FIP4	Forward	GGTGCATTGCCAAGGACTCA	129	NM_032932
	Reverse	GCCATCTAACAAACCCTTGGGC		
RNASE1	Forward	AGGCGCCGGAATATGACACA	73	NM_198232
	Reverse	GGACATCTACCAGGGGCTCG		
RPL23A	Forward	CGGGGCCATCCAGATTCAGT	76	NM_000984
	Reverse	GCCTTCAGACGCACTTCAGC		
RPS2	Forward	CTCCGCACCTGTGCCTAAGA	113	NM_002952
	Reverse	CATCAAAGGTGGCCTTGGCG		
RPS29	Forward	GGCACGGTCTGATCCGGAAA	122	NM_001032
	Reverse	GCCCCGGATAATCCTCTGAAGG		
SIGIRR	Forward	GGAGAGCCATCAGCTCCACC	102	AY358342
	Reverse	GGCACTGTAGTTTCGCGAGC		
SLC35F2	Forward	ACTCATTCCAGGTCCCAGAGC	71	NM_017515
	Reverse	ACTGTGGAGCACATAGCCAGT		
SLC36A1	Forward	GTGCATGCAGGCCCATGTTT	74	NM_078483
	Reverse	GCACGATGGCAACAGAGCAT		
STYXL1	Forward	ACTCTGAGCTCCGGAAAGGATG	143	NM_016086
	Reverse	ACTCCCATTTGGAACGGACAT		
